# The actin bundling protein Fascin is important for proper early development in Strongylocentrotus purpuratus embryos

**DOI:** 10.17912/micropub.biology.000717

**Published:** 2023-03-21

**Authors:** Michael D. Testa, Carolyn M. Remsburg, Jia L. Song

**Affiliations:** 1 University of Delaware, Newark, Delaware, United States

## Abstract

Fascin is a conserved protein that has been shown to modulate the cytoskeleton. Its role in early development remains unclear. After fertilization, embryos undergo rapid cell divisions, requiring the precise regulation of cytoskeleton to segregate chromosomes. Results indicate that Fascin is in the cell cortex, enriched in the perinuclear region of non-dividing blastomeres and on the mitotic spindle of dividing blastomeres of the early embryo. Loss-of-function of Fascin leads to a significant developmental delay or arrest, indicating that Fascin is important for proper early embryonic development.

**Figure 1. Knockdown of Fascin leads to delayed or arrested developmental progression:**
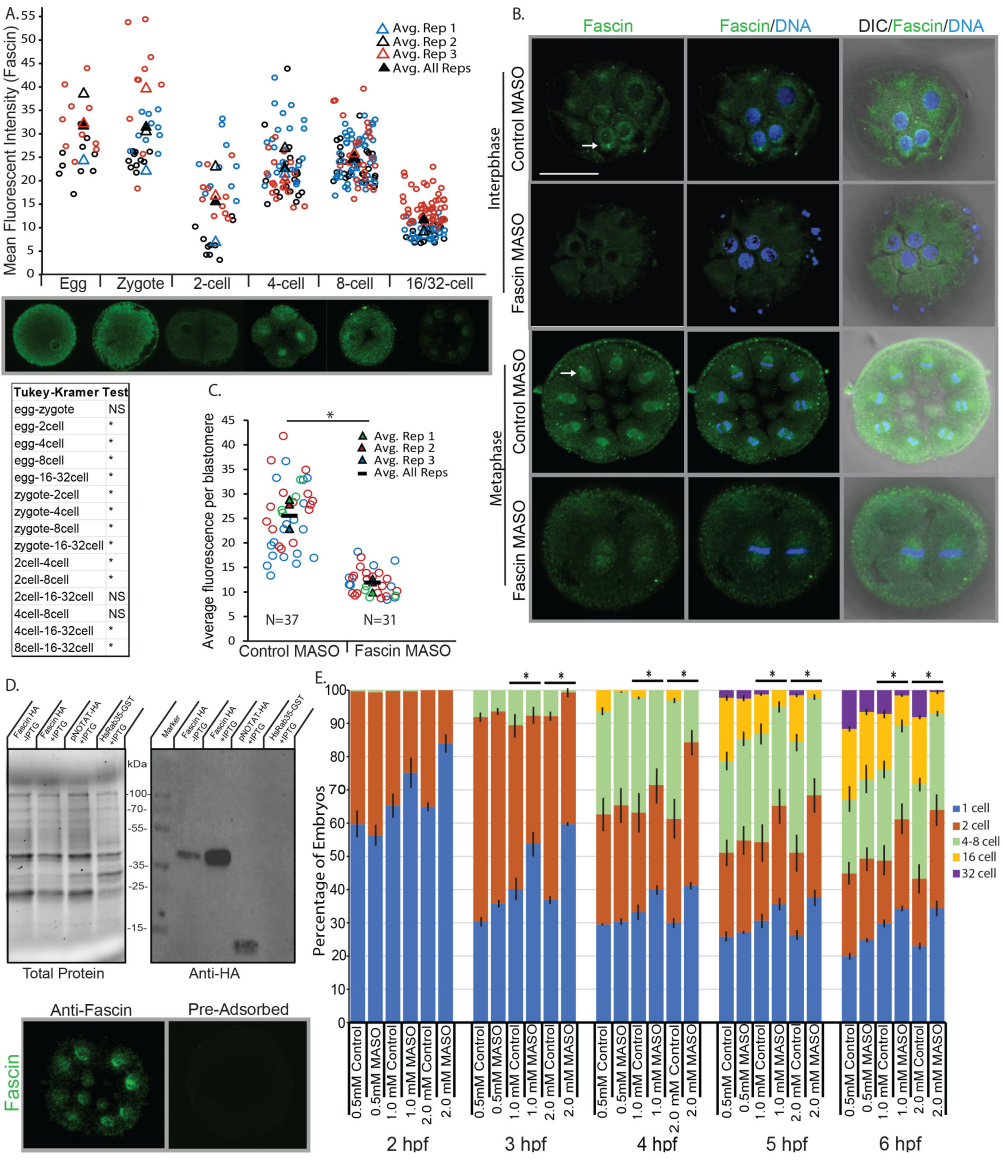
(A) Egg, zygote, 2-cell, 4-cell, 8-cell, 16-32-cell embryos were immunolabeled with Fascin antibody (green) to examine levels of Fascin protein through development. Tukey Kramer statistical test (Pollard et al., 2019) was used to analyze Fascin protein levels among various developmental stages. *denotes p<0.05. NS=not significant. (B) Control or Fascin MASO-injected (2 mM) embryos were immunolabeled with Fascin antibody (green) and counterstained with DAPI to label the DNA (blue). Fascin protein is localized perinuclearly in non-dividing blastomeres, enriched on the mitotic spindle in dividing blastomeres (arrows), and at the cell cortex. Fascin protein in Fascin MASO-injected embryos is significantly decreased compared to the control MASO-injected embryos. Scale bar = 50 µm; 3 biological replicates. (C) The level of Fascin protein in control and Fascin MASO-injected (2 mM) in individual blastomere of the 16-32 cell stage embryos was quantified with ImageJ, by using the average fluorescence per blastomere. Fascin protein is significantly decreased in embryos injected with the Fascin MASO compared to the control, demonstrating the efficacy of the Fascin knockdown. Circles represent individual measurements corresponding to each biological replicate. N=total number of blastomeres measured; 3 biological replicates; *denotes p<0.005 using a Student’s *t* -test comparing all measurements from 3 biological replicates. (D) The specificity of the Fascin antibody is tested with pre-adsorption assay. Fascin coding sequence is cloned into hemagglutinin (HA) and histidine (His)-tagged expression vector and transformed into C41 cells (Lucigen, Middleton, WI). Western blot indicates Fascin-HA-His tagged protein (37.98 kDa) is recognized with an anti-HA antibody. Bacterial lysates expressing Fascin-HA-His were used to conduct pre-adsorption tests. Embryos were immunolabeled with Fascin antibodies pre-adsorbed with lysate from bacteria expressing HA-tagged Fascin or human Rab35-GST. Embryos immunolabeled with pre-adsorbed Fascin antibodies have decreased Fascin (shown in green) compared to embryos immunolabeled with non-pre-adsorbed Fascin antibody. (E) Zygotes were injected with control or Fascin MASO at 0.5mM, 1mM, and 2mM. The percentage of embryos at a specific stage was recorded hourly from 2 to 6 hours post fertilization (hpf). Loss-of-function of Fascin results in developmental delay or arrest in early cleavage stage embryos in a dose-dependent manner. Error bars depict standard error of the mean (SEM). 3 biological replicates. *denotes p< 0.01 using Cochran-Mantel-Haenszel test (Mantel & Haenszel, 1959). N=number of embryos counted per timepoint.

## Description

Immediately after fertilization, metazoan embryos begin development with a series of rapid cell divisions (Siefert et al., 2015) . These early cleavage stage embryos cycle between mitosis and DNA synthesis with minimal gap phases (G1 and G2) (Siefert et al., 2015) . Mitosis is a highly regulated process, resulting in faithful segregation of the chromosomes. This process requires a highly ordered and coordinated assembly of the actin and microtubule (MT) cytoskeleton to form the mitotic spindle and undergo proper cytokinesis (Lancaster & Baum, 2014; Rizzelli et al., 2020) . Additionally, both spindle orientation and chromosome separation rely on actomyosin in the cell cortex providing a rigid scaffold to counteract the traction forces exerted by astral MTs pulling towards the spindle poles (Rizzelli et al., 2020) . In this study, we identified that Fascin plays an important role in early embryonic development, potentially through its ability to bundle actin and regulate microtubule polymerization (Bryan et al., 1993; Villari et al., 2015) , ultimately affecting the timely divisions of a rapidly dividing embryo.

Fascin, was first discovered in the actin-rich microvilli on the cell cortex of newly fertilized sea urchin eggs (Arshinoff et al., 2022; Otto et al., 1980) . It is an evolutionarily conserved protein found in organisms ranging from fruit fly, zebrafish, frogs, and mouse, with a canonical actin bundling function (Bryan et al., 1993; Lamb & Tootle, 2020) . Fascin is a 55 kDa protein consisting of four β-trefoil domains, all of which contain actin binding sites (Yang et al., 2013) . In zebrafish, Fascin has been shown to promote cell migration by forming filopodia in a subset of neural crest cells (Boer et al., 2015) . In *Drosophila* , the macrophage-like hemocytes in the developing embryo utilize Fascin to form filopodia (Zanet et al., 2009) , while the ovary migratory border cells require Fascin for their collective migration (Lamb et al., 2020) .

In addition to its actin-bundling function, recent data indicate that Fascin may also regulate microtubule dynamics (Villari et al., 2015) . In human cancer cell lines, Fascin has been shown to directly interact with microtubules through a binding site on the second β-trifold domain (Villari et al., 2015) . In these cells, a depletion of Fascin leads to a reduction in microtubule dynamics and increased microtubule stability (Villari et al., 2015) . *In vitro* co-sedimentation assays of purified Fascin and polymerized tubulin and fluorescence resonance energy transfer (FRET) between GFP–fascin and tubulin–mCherry in HeLa cells both indicate that Fascin is able to bind microtubules (Villari et al., 2015) . Additionally, microtubule binding contributes to Fascin-dependent control of focal adhesion dynamics and cell migration speed (Villari et al., 2015) . The ability of Fascin to bind and regulate actin and microtubules makes it an interesting protein to study in the context of early development, when the embryo is undergoing rapid cell divisions that require extensive cytoskeletal remodeling.

To understand the function of Fascin in cleavage stage embryos, we use the sea urchin *Strongylocentrotus purpuratus* . The sea urchin embryo is an excellent model, due to its high fecundity and ability to undergo external fertilization, resulting in transparent embryos which can withstand experimental manipulations (Mcclay, 2011). We used two Fascin antibodies developed against the human Fascin protein to examine the levels of Fascin protein throughout development. Results indicate that Fascin is maternally present and significantly decreased from the zygotic to 2-cell stage ( Fig. 1A ). The level of Fascin is then significantly increased from the 2-cell to 4-cell stage. These results suggest that post fertilization, maternal Fascin is degraded and newly translated from 2-cell to 4-cell stage, allowing for effective morpholino antisense-oligonucleotide (MASO)-based translational inhibition. Fascin immunolabeling reveals that Fascin has dynamic subcellular location: Fascin protein is enriched in the cell cortex, in perinuclear region of interphase blastomeres, and on the mitotic spindles of dividing blastomeres ( Fig. 1B ). Using Fascin MASO designed to anneal just upstream of the start codon of Fascin CDS (gene symbol FSCN1) (Arshinoff et al., 2022) , we demonstrated that embryos injected with the Fascin MASO exhibit a significant depletion of Fascin protein, compared to the control, indicating the effectiveness of the knockdown and specificity of the Fascin antibody ( Fig. 1C ). To further test the specificity of the Fascin antibody, we cloned the sea urchin Fascin coding sequence fused to a hemagglutinin-histidine-tagged plasmid (Fascin-HA-His) and expressed Fascin in bacteria ( Fig. 1D ). Results from the pre-adsorption assay indicate that the pre-adsorbed human Fascin antibody with the sea urchin Fascin recombinant protein have decreased Fascin immunostaining compared to the non-pre-adsorbed Fascin antibody immunostaining ( Fig. 1D ). Overall, these results indicate that the Fascin antibody made against human Fascin cross reacts with the sea urchin Fascin and demonstrate the effectiveness of the Fascin protein knockdown using Fascin translational blocking morpholino ( Fig. 1B -D).

The cell cycle-dependent enrichment of Fascin protein in the early embryo strongly suggests that it functions during early cleavage stage development. To examine Fascin’s function in greater detail, we conducted a time course experiment where we tabulated the percentage of embryos at a particular developmental stage in control and Fascin MASO-injected embryos. Results indicate that loss-of-function of Fascin leads to depleted Fascin and resulted in a significant delay or arrest of development in a dose-dependent manner ( Fig. 1E ). It is important to note that in control embryos, approximately 20-30% of embryos were arrested at the zygote stage. These arrested zygotes may result from suboptimal health of the animal or microinjection induced damage ( Fig. 1E ). Nevertheless, a significant delay in development is observed as early as 3 hpf in both 1 mM and 2 mM Fascin MASO-injected embryos ( Fig. 1E ). Since the maternal source of Fascin is decreased from zygotic to the 2-cell stage and increased from 2-cell to 4-cell stage ( Fig. 1A ), Fascin MASO can effectively prevent newly translated Fascin early in development. 5-6 hours into development, roughly 25% of control (2 mM) injected embryos have reached the 16-32 cell stage, whereas only about 7% of Fascin MASO (2 mM) knockdown embryos have developed to this stage. Fascin knockdown embryos appear to have cytokinetic defects ( Fig. 1B ) during mitosis, but more in-depth examination is needed. Thus, depletion of Fascin protein early in development leads to significant developmental delay or arrest in the sea urchin embryo. Taken together, these results indicate that Fascin is essential in early embryonic development and may promote normal, timely mitosis in early cleavage stage embryos.

Fascin has been found to localize inside the nucleus and to the nuclear periphery in *Drosophila* nurse cells and mammalian cells (Groen et al., 2015) . Previous work indicates that Fascin’s localization in and around the nucleus may be due to its regulation of nuclear actin and nucleolar morphology in both *Drosophila* nurse cells and mammalian cells (Groen et al., 2015) . Fascin has been shown to bundle critical nuclear actin rods (Kelpsch et al., 2016) needed in chromatin reorganization upon mitotic exit. During mitotic exit, newly formed cells undergo a profound reorganization of their nuclear content to re-establish an interphase nucleus, by forming transient nuclear actin rods to mediate the expansion of nuclear size and volume (Baarlink et al., 2017) . Thus, a potential role that Fascin plays in early development may be to mediate nuclear actin rods to facilitate mitotic exit. One potential explanation of our result is that a reduction of Fascin may lead to developmental delay and/or arrest, in part, due to the inability of Fascin to bundle nuclear actin rods needed for mitotic exit and subsequent cell divisions in a rapidly dividing embryo.

Our novel findings that Fascin protein localizes to the mitotic spindles and is important in early development lead to our hypothesis that through Fascin’s ability to bind to actin and microtubules, it may modulate dynamic cytoskeletal changes critical for early embryogenesis. Fascin has been shown to regulate actin assembly in mouse oocyte meiosis through its coordination with Daam1, a formin protein that can nucleate, elongate, and bundle actin (Lu et al., 2017) . A disruption in actin assembly upon Daam1 reduced expression prevented polar body emission during meiosis, as well as led to a decrease in Fascin protein (Lu et al., 2017) . This study suggests that Daam1 affects actin assembly during oocyte meiotic division possibly through its regulation of Fascin expression. Although meiosis and mitosis are different processes, they share similar hallmark of reorganizing the cytoskeleton to mediate division between cells (Brunet & Maro, 2005) . Thus, Fascin may potentially regulate actin to impact mitotic division, similar to how it regulates polar body emission during meiosis.

In the context of mitosis, cytoskeletal remodeling begins in prophase, when interphase microtubules are disassembled and a new population of shorter, more dynamic microtubules is nucleated from centrosomes (Niethammer et al., 2007) . Since Fascin protein localizes to the mitotic spindle and is important for early development ( Fig. 1E ), we propose that Fascin may directly mediate microtubule dynamics during mitosis. Thus, a knockdown of Fascin may perturb microtubule dynamics to negatively impact mitosis resulting in developmental delay ( Fig. 1E ). Further studies will need to be conducted to examine Fascin’s impact on the cytoskeleton.

In conclusion, we report that Fascin protein is localized to the cell cortex, perinuclearly in non-dividing blastomeres, and on the mitotic spindle of actively dividing blastomeres of the early embryo. Knockdown of Fascin leads to a developmental delay or arrest, potentially due to its ability to remodel actin and mediate microtubule dynamics in a dividing embryo. This study provides new insight into how Fascin may be necessary for proper cell division, and thus overall embryonic development.

## Methods

**Animals**

Adult purple sea urchins, *Strongylocentrotus purpuratus* , were obtained from Marinus Scientific, LLC (Lakewood, CA) and were maintained at 12°C in artificial sea water (ASW) made from distilled water and Instant Ocean©. Adults were induced to shed either through shaking or intracoelomic injection of 1 ml of 0.5 M KCl. Embryos were cultured at 12°C in filtered sea water (FSW) obtained from the Indian River Inlet (University of Delaware, Lewes, DE).

**Microinjections**

Microinjections were performed as described previously (Cheers and Ettensohn, 2004; Stepicheva and Song, 2014). All injection solutions contained 20% sterile glycerol and 2 mg/ml 10,000 MW neutral Texas Red or FITC anionic dextran (ThermoFisher, Waltham, MA) to mark the microinjected embryos. Injections were performed using the Pneumatic pump system (World Precision Instruments, Sarasota, FL). A vertical needle puller PL-10 (Narishige, Tokyo, Japan) was used to pull the injection needles (1 mm glass capillaries with filaments) (World Precision Instruments, Sarasota, FL). Fascin or control antisense morpholino oligonucleotide (MASO) were obtained from GeneTools (Philomath, OR). The Fascin (FSCN1) MASO (5′ ATCAACATATTTCACAATGCCTGCT 3′) was designed to be complementary to the 5′ region of the Fascin mRNA spanning before the beginning of the start codon. The sequence was determined to be unique to SpFascin by BLASTN of the sea urchin genome database. The control MASO (5′ CCTCTTACCTCAGTTACAATTTATA 3′) targets a human *β-globin* gene and is not complementary to the sea urchin genome (as determined by BLASTN against the sea urchin genome database). The Fascin and control MASOs were injected at final concentrations of 0.5mM, 1.0mM and 2.0mM. We monitored the development of control or Fascin MASO-injected embryos, by tabulating the number of embryos that reached a particular developmental stage on an hourly basis until the 32-cell stage. We categorized our developmental stages at a range of 1-cell (undivided zygote), 2-cell, 4/8-cell, 16-cell, and 32-cell embryos. After it was determined that injection of 2mM Fascin MASO resulted in approximately 50% normal developing embryos, that dosage was used for Fascin immunofluorescence.

**Immunofluorescence**

To examine the efficacy of Fascin knockdown with Fascin MASO, both control and MASO-injected embryos were immunolabeled with two Fascin monoclonal antibodies (ECM Biosciences LLC, Versailles, KY; Proteintech Group, Inc, Rosemont, IL). Fascin antibodies were developed against the human peptide sequence. After microinjection, embryos were fixed with ice cold 100% methanol for 10 minutes. 16-32 cell embryos were washed with PBST (1x PBS, 0.1% TritonX-100) for 10 minutes each, and then blocked with PBST and 4% sheep serum for 1 hour at room temperature. Embryos were incubated with Fascin primary antibody (1:100 in PBST and 4% sheep serum) for 24hours at 4°C. Embryos were then washed three times in PBST and incubated in Alexa 488 conjugated goat-anti-mouse antibody (ThermoFisher, Waltham, MA) at a 1:300 dilution in 4% sheep serum in PBST for 1 h at room temperature. Embryos were washed three times with PBST, and counter stained with NucBlue™ (DAPI) DNA stain (ThermoFisher, Waltham, MA) at 1 drop per 500µL dilution, according to the product instructions. Embryos were then imaged using Zeiss LSM 880 confocal microscope with Zen software. Single digital image or the maximum intensity projections of Z-stack of images were acquired with Zen software. Images were processed using Adobe Photoshop and Illustrator (Adobe, San Jose, CA). Measurements for average fluorescent intensity of Fascin signals were acquired from a range of Z-stack images based on DNA as a reference point using Zen software and quantitated with ImageJ (Schneider, Rasband and Eliceiri, 2012).

**Protein Expression/Western Blotting**

We cloned Fascin CDS upstream of a HA and 6x-poly histidine into pNOTAT expression vector (Nagahara et al., 1998) , with the expected size of a ⁓38 kDa protein, herein referred to as Fascin-HA-His. Sea urchin Fascin-HA-His was transformed into C41 competent cells (Lucigen, Middleton, WI). For negative controls, C41 competent cells were transformed with *Homo sapiens* Rab35-GST-pGEX6P-1 and the empty pNOTAT vector. All transformed cells were induced with 1mM IPTG for protein expression at 37°C for 4.5 hours. After induction, 1mL of bacterial lysate was spun down at 15,871 x *g* for 20 minutes at 4°C. Supernatant was removed and discarded. To the bacterial pellets, 100µL of Laemmli buffer (4% SDS, 20% glycerol, 0.004% bromophenol blue, 0.125M Tris-Cl, pH 6.8) and 5% 2-Mercaptoethanol (Thermo Fisher Scientific, Waltham, MA) were added. Samples were vortexed to resuspend the pellet, boiled at 100°C, and spun down at 15,871 x *g* for 5 minutes at 4°C. 20µL of samples were run on a 12% SDS-PAGE gel made by following instructions from the manufacturer (BioRad, Philadelphia, PA). Total protein run on the gel was captured using BioRad Chemidoc Gel Imager (BioRad, Philadelphia, PA). The protein gel was transferred to PVDF membrane (BioRad, Philadelphia, PA) using a semi-dry transfer system (BioRad, Philadelphia, PA). After transfer, the blot was blocked in 5% bovine serum albumin (BSA) (Research Products International, Mount Prospect, IL) in TBST (20mM Tris-base, 150mM NaCl, 50mM KCI, 0.2% Tween-20, pH 7.6) for 2 hours at room temperature on a rocker. Blots were then incubated in monoclonal anti-HA antibody at a dilution of 1:5000 (ThermoFisher, Waltham, MA) in 5% BSA/TBST for 2 hours at 4°C on a rocker. After primary antibody incubation, blots were washed 5 times in TBST with vigorous shaking at room temperature for 10 minutes each wash. Blots were then incubated in secondary rabbit anti-mouse IgG HRP (Thermo Fisher Scientific, Waltham, MA) at a dilution of 1:10,000 in 5% BSA and TBST for 1 hour at room temperature on a rocker. After washing 5 times with TBST, the blot was exposed with SuperSignal West Pico Chemiluminescent Substrate (Thermo Fisher Scientific, Waltham, MA) and imaged with BioRad Chemidoc Gel Imager (BioRad, Philadelphia, PA).

**Pre-adsorption assay of Fascin**

A pre-adsorption assay was performed to test the specificity of the Fascin antibody. The Fascin antibody was pre-adsorbed in blocking buffer (PBST- 0.1% Triton in 4% sheep serum) or with bacterial lysate expressing the sea urchin Fascin protein coated on the PVDF 0.5 cm x 0.5 cm membranes (BioRad, Philadelphia, PA) in Eppendorf tubes for one night at 4°C. Fascin antibody pre-adsorbed with bacterial lysate with or without sea urchin Fascin was used in immunolabeling experiments. Embryos were washed with PBST and incubated with goat anti-rabbit Alexa 488 in blocking buffer for 1 hour at room temperature and counterstained with DAPI. Single slice images were acquired with Zeiss LSM 880 scanning confocal microscope (Carl Zeiss Incorporation, White Plains, NY).

**Fascin protein quantification through development**

Fascin protein through development was quantified using immunofluorescence. Eggs, zygotes, 2-cell, 4-cell, 8-cell, and 16-32 cell embryos were collected and fixed with 100% methanol on ice for 10 minutes. Eggs, zygotes, and embryos were washed with PBST (1x PBS, 0.1% TritonX-100) for 10 minutes each, and then blocked with PBST and 4% sheep serum for 1 hour at room temperature. Embryos were incubated with Fascin primary antibody (1:100 in PBST and 4% sheep serum) for 24 hours at 4°C. Embryos were then washed three times in PBST and incubated in Alexa 488 conjugated goat-anti-mouse antibody (ThermoFisher, Waltham, MA) at a 1:300 dilution in 4% sheep serum in PBST for 1 hour at room temperature. Embryos were washed three times with PBST, and counter stained with NucBlue™ (DAPI) DNA stain (ThermoFisher, Waltham, MA) at 1 drop per 500µL dilution, according to the product instructions.

**ImageJ Analysis**

To quantitatively analyze the efficacy of the Fascin knockdown with MASO, individual blastomeres (non-dividing) of both control and Fascin MASO-injected 32-cell embryos were analyzed after immunolabeling with Fascin antibody. Non-dividing blastomeres were selected and orthological projections of the green (Alexa 488) channel of +/- 4 µm from the midpoint were compiled and exported from Zen as a TIFF (Tag Image File Format). It is important to note that the highest fluorescent intensity for DAPI was used as a midpoint. These images were analyzed in ImageJ (Schneider, Rasband and Eliceiri, 2012). A region spanning the area of each blastomere was selected and the mean fluorescence intensity (MFI) was measured. Background signal taken from the outside of the embryo was subtracted from the fluorescent reading within the blastomere for normalization. The same image analysis was used to quantify the levels of Fascin protein throughout development.

## Reagents

**Reagents**

**Table:** 

**ANTIBODY/STAIN**	**ANIMAL AND CLONALITY**	**DESCRIPTION**	**AVAILABLE FROM**
**Fascin (55K2 clone)**	Mouse monoclonal	Clone (55K2) was generated from full-length human fascin purified from HeLa cells. IgG1 Isotype	ECM Biosciences LLC(Versailles, KY)
**Fascin mouse mcAb**	Mouse monoclonal	Unconjugated Mouse / IgG2a	Proteintech Group, Inc, (Rosemont, IL)
**Monoclonal Anti-HA**	Monoclonal	IgG	Thermo Fisher Scientific (Waltham, MA)
**NucBlue™ Fixed Cell ReadyProbes™ Reagent (DAPI)**	N/A	Nuclear counterstain for fixed cells that emits blue fluorescence when bound to DNA.	ThermoScientific (Waltham, MA)

**Table:** 

**MORPHOLINO ANTISENSE OLIGONUCLEOTIDE**	**DESCRIPTION**	**AVAILABLE FROM**
**Fascin MASO 5mM Stock**	Fascin MASO (5′ ATCAACATATTTCACAATGCCTGCT 3′) was designed to be complementary to the 5′ region of the *Fascin* mRNA spanning before the beginning of the start codon.	GeneTools (Philomath, OR)
**Control MASO 5mM Stock**	The control MASO (5′ CCTCTTACCTCAGTTACAATTTATA 3′) designed to target a human *β-globin* gene and is not complementary to the sea urchin genome.	GeneTools (Philomath, OR)

## References

[R1] Arshinoff BI, Cary GA, Karimi K, Foley S, Agalakov S, Delgado F, Lotay VS, Ku CJ, Pells TJ, Beatman TR, Kim E, Cameron RA, Vize PD, Telmer CA, Croce JC, Ettensohn CA, Hinman VF (2022). Echinobase: leveraging an extant model organism database to build a knowledgebase supporting research on the genomics and biology of echinoderms.. Nucleic Acids Res.

[R2] Baarlink C, Plessner M, Sherrard A, Morita K, Misu S, Virant D, Kleinschnitz EM, Harniman R, Alibhai D, Baumeister S, Miyamoto K, Endesfelder U, Kaidi A, Grosse R (2017). A transient pool of nuclear F-actin at mitotic exit controls chromatin organization.. Nat Cell Biol.

[R3] Boer EF, Howell ED, Schilling TF, Jette CA, Stewart RA (2015). Fascin1-dependent Filopodia are required for directional migration of a subset of neural crest cells.. PLoS Genet.

[R4] Brunet S, Maro B (2005). Cytoskeleton and cell cycle control during meiotic maturation of the mouse oocyte: integrating time and space.. Reproduction.

[R5] Bryan J, Edwards R, Matsudaira P, Otto J, Wulfkuhle J (1993). Fascin, an echinoid actin-bundling protein, is a homolog of the Drosophila singed gene product.. Proc Natl Acad Sci U S A.

[R6] Groen CM, Jayo A, Parsons M, Tootle TL (2015). Prostaglandins regulate nuclear localization of Fascin and its function in nucleolar architecture.. Mol Biol Cell.

[R7] Kelpsch DJ, Groen CM, Fagan TN, Sudhir S, Tootle TL (2016). Fascin regulates nuclear actin during Drosophila oogenesis.. Mol Biol Cell.

[R8] Lamb MC, Anliker KK, Tootle TL (2020). Fascin regulates protrusions and delamination to mediate invasive, collective cell migration in vivo.. Dev Dyn.

[R9] Lamb MC, Tootle TL (2020). Fascin in Cell Migration: More Than an Actin Bundling Protein.. Biology (Basel).

[R10] Lancaster OM, Baum B (2014). Shaping up to divide: coordinating actin and microtubule cytoskeletal remodelling during mitosis.. Semin Cell Dev Biol.

[R11] Lu Y, Zhang Y, Pan MH, Kim NH, Sun SC, Cui XS (2017). Daam1 regulates fascin for actin assembly in mouse oocyte meiosis.. Cell Cycle.

[R12] MANTEL N, HAENSZEL W (1959). Statistical aspects of the analysis of data from retrospective studies of disease.. J Natl Cancer Inst.

[R13] McClay DR (2011). Evolutionary crossroads in developmental biology: sea urchins.. Development.

[R14] Nagahara H, Vocero-Akbani AM, Snyder EL, Ho A, Latham DG, Lissy NA, Becker-Hapak M, Ezhevsky SA, Dowdy SF (1998). Transduction of full-length TAT fusion proteins into mammalian cells: TAT-p27Kip1 induces cell migration.. Nat Med.

[R15] Niethammer P, Kronja I, Kandels-Lewis S, Rybina S, Bastiaens P, Karsenti E (2007). Discrete states of a protein interaction network govern interphase and mitotic microtubule dynamics.. PLoS Biol.

[R16] Otto JJ, Kane RE, Bryan J (1980). Redistribution of actin and fascin in sea urchin eggs after fertilization.. Cell Motil.

[R17] Pollard DA, Pollard TD, Pollard KS (2019). Empowering statistical methods for cellular and molecular biologists.. Mol Biol Cell.

[R18] Rizzelli F, Malabarba MG, Sigismund S, Mapelli M (2020). The crosstalk between microtubules, actin and membranes shapes cell division.. Open Biol.

[R19] Siefert JC, Clowdus EA, Sansam CL (2015). Cell cycle control in the early embryonic development of aquatic animal species.. Comp Biochem Physiol C Toxicol Pharmacol.

[R20] Villari G, Jayo A, Zanet J, Fitch B, Serrels B, Frame M, Stramer BM, Goult BT, Parsons M (2015). A direct interaction between fascin and microtubules contributes to adhesion dynamics and cell migration.. J Cell Sci.

[R21] Yang S, Huang FK, Huang J, Chen S, Jakoncic J, Leo-Macias A, Diaz-Avalos R, Chen L, Zhang JJ, Huang XY (2012). Molecular mechanism of fascin function in filopodial formation.. J Biol Chem.

[R22] Zanet J, Stramer B, Millard T, Martin P, Payre F, Plaza S (2009). Fascin is required for blood cell migration during Drosophila embryogenesis.. Development.

